# Diseases of Twins

**Published:** 1907-11-23

**Authors:** J. Lionel Tayler

**Affiliations:** 8 Adys Lawn, St. Paul's Avenue, Willesden Green, London, N.W.


### DISEASES OF TWINS.

SIR,—Will you kindly permit me to make use of the columns of your medical journal under the following circumstances? On re-reading Francis Galton's "History of Twins," published in his work "Inquiries into Human Faculty," it occurred to me that, as he has proved the greater strength of Nature over nurture in this investigation, the same material, slightly modified, would be able to conclusively establish the relative importance of the factors of individual constitution and specific invading organisms in the production of disease. Accordingly I had, as a beginning, two hundred circulars and addressed return-envelopes printed, and subsequently despatched a little over fifty of them, to test the kind of response I was likely to meet with. I had replies from over forty per cent., and although I took the precaution of sending only to general practitioners of more than ten years' standing, the evidence obtained was decidedly disappointing. I had imagined, as twin births are said to occur once in about every ninety maternity cases, that most practitioners who had been in practice for a few years would have had some experience on this subject, even allowing for the fact that twin children frequently die.

To my surprise, no less than forty-five per cent. of my replies stated that no experience had so far been obtained; eight to ten per cent. apparently do not practice midwifery, or discourage it, and have not attended twins at any later age period; and only forty-five per cent. have had any experience, however meagre. Of these most appear to have had a few maternity cases, though the answers are not always clear on this point; in addition twenty-five per cent. have once attended twin children, and thirty per cent. once adults. I am not quite certain whether one of these latter did not attend adult twins twice. The number of replies is, of course, far too small to make any generalisation upon the matter. A practitioner may possibly be unaware that he is treating a twin when the pair are separated. Still, allowing for this, the scarcity of material is remarkable. The explanation may possibly be due to an error in assuming that hospital statistics, drawn from the poorest citizens, apply equally to all classes.

In any case, it is evident that I must appeal to a much larger circle of medical men than can be done by any method of private correspondence, in order to obtain the necessary information.

Would any of your readers, therefore, who have in their possession evidence on the points mentioned below, kindly communicate with the writer, whose address is appended? Twins, if they are true twins, are usually of the same sex, and are most frequently either much alike or much unlike.

(1) Of like twins of the same sex, at any age period, have you any medical experience of their suffering simultaneously or independently from like or unlike diseases in like or unlike (separate) surroundings? (2) Of unlike twins of the same sex, at any age period, have you any medical experience of their suffering simultaneously or independently from like or unlike diseases in like or unlike (separate) surroundings?

Please name specifically all diseases referred to, as this point is very important. Any additional information on the subject would be welcomed, and all remarks will, of course, be treated confidentially. I would be greatly obliged if Continental journals would insert this letter in their issues.

Thanking you and your readers in anticipation for your kindness in considering my letter,

I am, Sir, yours faithfully,

J. LIONEL TAYLER.

8 Adys Lawn, St. Paul's Avenue,
Willesden Green, London, N.W.

